# 184. Incidence and Clinical Outcomes of RSV and Influenza in Solid Organ Transplant Recipients

**DOI:** 10.1093/ofid/ofaf695.062

**Published:** 2026-01-11

**Authors:** Vivian Bui Le, Helene Høgsbro Thygesen, Michael Perch, Søren Scwartz Sørensen, Kasper Rossing, Nicolai Aagaard Schultz, Jens Lundgren, Marie Helleberg

**Affiliations:** Centre of Excellence for Health, Immunity and Infections - CHIP, Copenhagen University Hospital, Rigshospitalet, Denmark, Copenhagen, Hovedstaden, Denmark; Rigshospitalet, Copenhagen, Hovedstaden, Denmark; University of Copenhagen, Copenhagen, Hovedstaden, Denmark; Rigshospitalet, Copenhagen, Hovedstaden, Denmark; Rigshospitalet, Copenhagen, Hovedstaden, Denmark; Rigshospitalet, Copenhagen, Hovedstaden, Denmark; Rigshospitalet, University of Copenhagen, Copenhagen, Hovedstaden, Denmark; Rigshospitalet, Copenhagen University Hospital, Copenhagen, Hovedstaden, Denmark

## Abstract

**Background:**

Solid organ transplant (SOT) recipients are due to their immunosuppressed state susceptible to serious disease caused by respiratory tract infections with influenza or respiratory syncytial virus (RSV). While there is extensive knowledge on influenza among SOT recipients, data on how RSV affect this population remain limited and inconsistent. It is unknown whether severity and outcomes differ between influenza and RSV.

**Figure:**
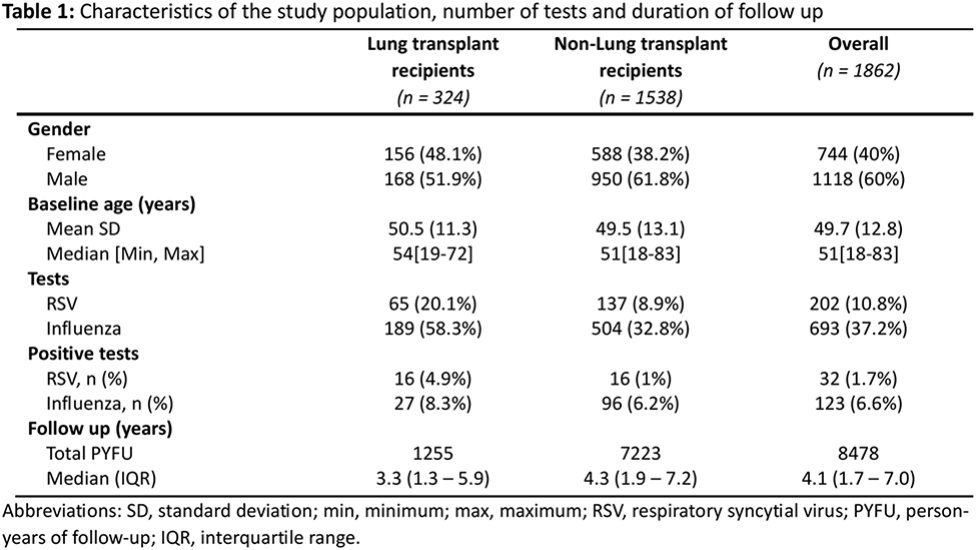

**Figure:**
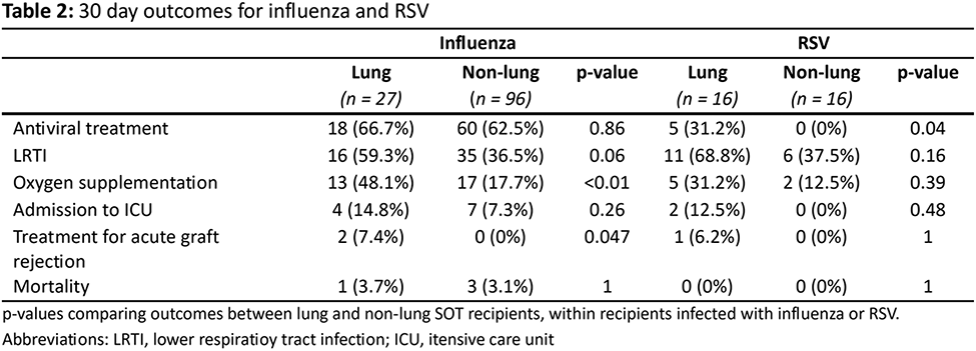

**Methods:**

All SOT recipients at Copenhagen University Hospital, Denmark, aged >18 years and transplanted in the study period 1^st^ January 2010 to 28^th^ February 2021 were included. The cumulative incidence rate of tests and positive tests were analyzed from date of transplantation until death, re-transplantation, emigration, or end of study. To examine associations between influenza/RSV and mortality we estimated mortality rates (MR) in time periods before infection (including patients who never tested positive), 0-180 days and > 180 after infection. MR ratios were estimated using Poisson regression analyses, adjusted for age, gender, time after transplant, and transplantation type.

**Figure:**
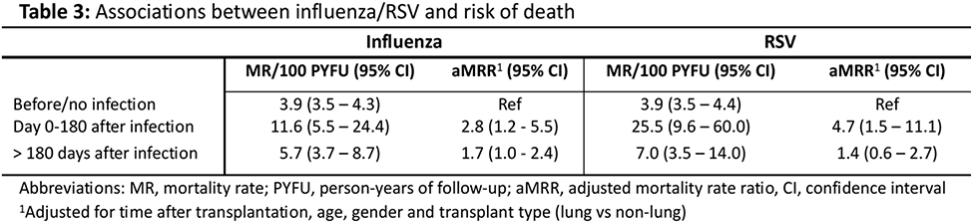

**Figure:**
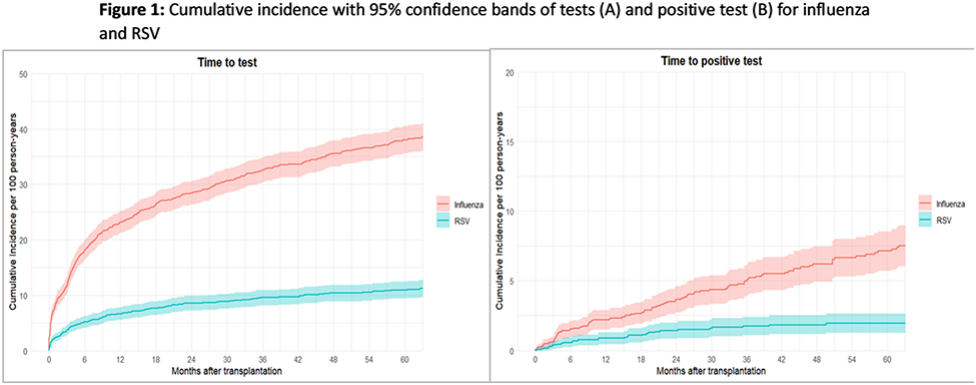

**Results:**

A total of 1862 SOT recipients were included (Table 1), of whom 693 (37.2%) and 202 (10.8%) were tested for influenza and RSV, respectively (Figure 1); test positive rates were 17.7% for influenza and 15,8% for RSV. There was a tendency towards more severe outcomes for lung versus for non-lung SOT recipients in the first 30 days after infection, but most comparisons did not reach statistical significance (Table 2). Influenza and RSV were associated with an approximately 3-5 fold increased mortality the first 6 months following infection (Table 3).

**Conclusion:**

Testing was more frequent for influenza than RSV, but test-positive rates were similar suggesting underdiagnosis of RSV. Both influenza and RSV were associated with significantly increased mortality the first 6 months following infection. These findings suggest importance of broader testing for RSV and prevention efforts.

**Disclosures:**

All Authors: No reported disclosures

